# Time-Varying Low-Frequency Electromagnetic Fields and Endothelial Angiogenesis: From Early In Vitro Discoveries to Emerging Mechanisms

**DOI:** 10.3390/pathophysiology33030057

**Published:** 2026-08-10

**Authors:** Linghong Li, Wayne F. Patton

**Affiliations:** Bioelectromagnetic Interaction Laboratory, Department of Physics, Florida Polytechnic University, Lakeland, FL 33805, USA; echinasun@gmail.com

**Keywords:** low-frequency electromagnetic fields, pulsed electromagnetic fields, angiogenesis, endothelial cells, macropinocytosis, vesicular trafficking, calcium signaling, vascular remodeling

## Abstract

Background/Objectives: Time-varying low-frequency electromagnetic fields (LF-EMFs), including pulsed electromagnetic fields (PEMFs) and sinusoidal low-frequency electromagnetic fields, have been investigated as modulators of tissue repair and vascular remodeling. Although numerous studies report pro-angiogenic effects in endothelial systems, the underlying mechanisms remain incompletely understood. This review synthesizes evidence for LF-EMF-induced endothelial angiogenesis and evaluates emerging mechanistic explanations. Methods: Literature was identified through searches of PubMed, Web of Science, and Google Scholar through March 2026. Peer-reviewed studies emphasizing in vitro endothelial angiogenesis models were prioritized, with selected in vivo studies included when relevant to mechanistic interpretation. The review was conducted as a narrative synthesis rather than a systematic review or meta-analysis. Results: Experimental evidence indicates that LF-EMF exposure can enhance endothelial proliferation, migration, sprouting, tube formation, and survival across diverse model systems. Reported mechanisms include modulation of growth factor signaling, calcium-dependent pathways, nitric oxide production, cytoskeletal remodeling, and mechanotransduction. Reexamination of early ultrastructural studies suggests that membrane ruffling, vacuole formation, and vesicular structures observed following LF-EMF exposure may be interpreted within the framework of macropinocytosis and vesicular trafficking, providing a potential mechanistic link between electromagnetic stimulation and endothelial morphogenesis. Conclusions: Current evidence supports the conclusion that LF-EMFs can modulate key endothelial processes involved in angiogenesis and vascular remodeling. Membrane trafficking and macropinocytosis provide a biologically plausible framework linking historical observations with contemporary endothelial cell biology, although direct experimental validation is needed. Future progress will require standardized exposure paradigms and mechanistic studies using physiologically relevant angiogenesis models.

## 1. Introduction

Time-varying low-frequency electromagnetic fields (LF-EMFs) have been widely investigated as therapeutic tools for tissue repair and, at times, scrutinized as potential public health concerns, making the establishment of definitive biological functions one of the more challenging and controversial topics in biophysics [1,2,3]. For the purposes of this review, the term low-frequency electromagnetic fields (LF-EMFs) refers collectively to time-varying electromagnetic field exposures below 300 Hz, encompassing both the internationally defined extremely low frequency (ELF; 3–30 Hz) and super low frequency (SLF; 30–300 Hz) bands, including both pulsed electromagnetic fields (PEMFs) and continuous sinusoidal fields. LF-EMFs have been investigated for more than five decades for their ability to modulate cellular behavior and promote tissue repair [4]. Early clinical applications focused primarily on orthopedic indications, particularly enhancement of bone healing, which stimulated broader investigation into electromagnetic field effects on other cell types, including endothelial cells [5,6].

Angiogenesis is a tightly regulated multistep process essential during development, tissue repair, and numerous pathological conditions. A growing body of experimental evidence indicates that LF-EMF exposure can influence multiple stages of endothelial angiogenesis, including proliferation, migration, sprouting, and capillary-like tube formation [7]. Formation of patent endothelial lumens requires coordinated extracellular matrix remodeling, cytoskeletal organization, and assembly of specialized signaling complexes that regulate three-dimensional tube formation [8]. These coordinated processes underlying new blood vessel formation suggest that LF-EMFs may modulate endothelial phenotype and morphogenesis rather than acting solely as mitogenic stimuli. However, despite decades of investigation, the cellular and molecular mechanisms linking electromagnetic field stimulation to endothelial remodeling remain incompletely understood.

Several prior reviews have summarized the broader effects of LF-EMFs on endothelial biology and tissue repair [2,9]. Rather than providing another comprehensive survey, this narrative mechanistic review focuses specifically on in vitro endothelial angiogenesis models that permit mechanistic interpretation of LF-EMF responses. Particular emphasis is placed on reexamining early ultrastructural observations through the lens of contemporary membrane trafficking biology. To our knowledge, this is the first review to interpret LF-EMF-induced endothelial ultrastructural changes in the context of macropinocytosis and vesicular trafficking, suggesting a potential mechanistic connection between electromagnetic field stimulation, endothelial remodeling, and vascular morphogenesis. Accordingly, the objectives of this review are to synthesize evidence for LF-EMF-induced endothelial angiogenesis in vitro, evaluate proposed cellular and molecular mechanisms, and develop a mechanistic framework linking historical observations with emerging concepts of membrane trafficking and endothelial morphogenesis. Understanding how LF-EMFs influence endothelial remodeling is relevant not only to regenerative medicine but also to pathological angiogenesis associated with wound repair, ischemic disease, inflammation, and tumor vascularization.

## 2. Literature Search Strategy

Literature was identified through searches of PubMed, Web of Science, and Google Scholar through March 2026 using combinations of the terms “electromagnetic field,” “pulsed electromagnetic field,” “low-frequency electromagnetic field,” “endothelial cell,” “angiogenesis,” “tube formation,” “migration,” “sprouting,” and “vascular morphogenesis.” Additional relevant publications were identified through citation tracking of key articles and recent reviews.

Priority was given to peer-reviewed original research articles investigating the effects of LF-EMFs on endothelial angiogenic responses, particularly in vitro studies evaluating endothelial proliferation, migration, sprouting, tube formation, signaling pathways, and related mechanistic endpoints. Landmark studies that established the historical development of the field were included alongside recent investigations to provide a comprehensive perspective on scientific progress. Selected in vivo studies were incorporated when they provided important mechanistic or physiological context for endothelial angiogenesis, whereas review articles were used primarily for historical background and conceptual framing rather than as sources of primary experimental evidence.

Publications written in English were included, together with selected publications in Chinese, reflecting the native fluency of the first author and allowing direct evaluation of studies that were particularly relevant to the scope of the review. Studies focusing exclusively on non-endothelial cell types or biological outcomes unrelated to angiogenesis were generally excluded unless they contributed directly to mechanistic interpretation or to the broader understanding of bioelectromagnetic interactions.

Because the objective of this work was to synthesize historical developments, critically evaluate mechanistic evidence, and identify emerging conceptual frameworks, the review was conducted as a narrative review rather than a formal systematic review or meta-analysis.

## 3. Evidence for LF-EMF-Induced Endothelial Angiogenesis

### 3.1. In Vitro Endothelial Angiogenesis Models

In vitro responsiveness of endothelial angiogenesis to LF-EMFs has been demonstrated across a consistent set of canonical model systems, with convergent phenotypic outcomes. The studies summarized in Table 1 differ considerably with respect to endothelial cell models, waveform characteristics, magnetic field intensity, exposure duration and biological endpoints. However, as summarized in Table 1, angiogenic responses have been observed across diverse endothelial cell models exposed to both pulsed and sinusoidal LF-EMFs, suggesting that endothelial angiogenic responses have been observed across a broad range of experimental conditions despite considerable methodological heterogeneity. The most widely used assay has been the extracellular matrix–based tube formation model, in which endothelial cells organize into capillary-like networks. LF-EMF exposure, including both pulsed and sinusoidal waveforms, has been shown to increase tube length, branching, and overall network complexity [10,11,12].

Complementary proliferation and migration assays, including BrdU incorporation, scratch wound healing, and endothelial migration (e.g., Transwell^®^ chamber, Corning Incorporated, Corning, NY, USA), further demonstrate that LF-EMF exposure enhances endothelial expansion and motility, often in association with VEGF-dependent signaling pathways [10,11]. In addition, survival-based models indicate that LF-EMF exposure reduces apoptosis under stress conditions such as hypoxia or serum deprivation, supporting endothelial persistence during angiogenic remodeling [12].

More structurally complex systems, including three-dimensional collagen or fibrin gel cultures and endothelial spheroid sprouting assays, extend these findings by demonstrating enhanced sprout formation and network development in matrix-embedded environments under LF-EMF stimulation [11,21]. Finally, co-culture and biomaterial-based models incorporating endothelial cells with mesenchymal or osteogenic lineages show increased expression of angiogenic markers, including VEGF and CD31, along with improved vascular-like organization following LF-EMF exposure [22].

Collectively, these in vitro studies indicate that LF-EMF exposure can enhance key endothelial angiogenic processes under diverse experimental conditions, although the magnitude of these responses varies with exposure parameters and experimental model.

### 3.2. Proposed Physical Basis for LF-EMF-Induced Endothelial Signaling

Although numerous studies have demonstrated that low-frequency electromagnetic fields (LF-EMFs) modulate endothelial proliferation, migration, sprouting, tube formation, and angiogenesis, the initial physical interaction by which weak LF-EMFs are detected by cells remains incompletely understood. Unlike ionizing radiation, LF-EMFs lack sufficient photon energy to directly break chemical bonds and, under therapeutic exposure conditions, produce negligible tissue heating. Consequently, current research has focused on identifying biophysical mechanisms capable of coupling weak LF-EMFs to endogenous cellular signaling pathways rather than causing direct molecular damage [23,24].

Proposed mechanisms can be considered at three hierarchical levels. At the molecular level, LF-EMFs have been hypothesized to influence ion dynamics, radical chemistry, or other magnetically sensitive molecular processes. At the supramolecular level, effects have been proposed on plasma membrane organization, ion channel activity, receptor clustering, cytoskeletal architecture, mitochondrial function, and membrane trafficking, all of which may amplify weak physical stimuli into measurable biochemical responses [24]. These primary interactions may subsequently converge at the systems level, where coordinated activation of intracellular signaling pathways regulates endothelial proliferation, migration, sprouting, lumen formation, and vascular morphogenesis. This hierarchical framework emphasizes that multiple physical mechanisms may contribute to a common biological response rather than implying a single universal initiating event.

Several candidate physical mechanisms have been proposed over the past four decades. Early hypotheses emphasized resonance phenomena involving biologically important ions, particularly calcium, including the ion cyclotron resonance (ICR) model [25]. Although these models stimulated extensive experimental investigation, subsequent theoretical analyses questioned whether such resonance phenomena can be sustained within the highly damped intracellular environment, and they are now generally regarded as historically important hypotheses rather than established mechanisms [26]. Radical-pair mechanisms, modulation of membrane-associated signaling complexes, and nitric oxide-dependent signaling have emerged as complementary areas of investigation [26,27]. While no single physical mechanism has achieved universal acceptance, there is increasing agreement that diverse initiating events may converge on common downstream signaling pathways. The following sections therefore focus on these experimentally supported endothelial signaling mechanisms, including calcium signaling, nitric oxide production, growth factor signaling, and membrane trafficking, which collectively provide the strongest mechanistic framework for understanding LF-EMF-induced angiogenesis. Accordingly, rather than emphasizing any single initiating physical mechanism, this review focuses on the downstream endothelial signaling pathways that have been experimentally demonstrated to mediate LF-EMF-induced angiogenic responses.

### 3.3. Downstream Signaling Pathways Underlying LF-EMF-Induced Endothelial Angiogenesis

Across more than four decades of investigation, studies of LF-EMFs, including both pulsed and sinusoidal exposures, have revealed consistent endothelial response patterns. LF-EMF exposure has been repeatedly associated with increased endothelial proliferation, enhanced migration, stimulation of sprouting behavior, and promotion of capillary-like tube formation [10,11,13,14].

Studies in non-endothelial cell types have reported LF-EMF-induced changes in calcium regulation, cytoskeletal organization, migration, proliferation, and oxidative signaling. These findings provide useful mechanistic hypotheses but cannot be assumed to predict angiogenic responses because EMF effects depend on cell lineage, differentiation state, exposure conditions, and biological context. This distinction was illustrated by Chung-Welch et al. (1989), who compared BPMEC with closely related pericardial mesothelial cells [13]. Although both are mesoderm-derived simple squamous epithelial cells with similar morphology and cytoskeletal protein profiles, only the endothelial cells underwent capillary-like tubule formation following LF-EMF exposure. The mesothelial cells exhibited no comparable morphogenetic response. This study demonstrates that while LF-EMFs may influence generalized cellular behaviors common to many cell types, angiogenesis requires activation of an endothelial-specific differentiation program that cannot be inferred from changes in proliferation or migration alone. Accordingly, the mechanistic discussion that follows emphasizes evidence obtained directly from vascular endothelial models.

Mechanistically, calcium signaling may represent one of the earliest convergent events linking LF-EMF exposure to downstream endothelial remodeling processes. Calcium signaling emerges as a plausible upstream regulator capable of coordinating multiple angiogenic processes, including cytoskeletal remodeling, nitric oxide production, and growth factor responsiveness [9,11,12,15,17,18]. In parallel, several studies indicate that LF-EMF exposure enhances endothelial sensitivity to pro-angiogenic cues such as VEGF and FGF-2, rather than acting as a primary mitogenic stimulus [11,12,15,17,18]. Emerging evidence suggests that several of these signaling pathways may converge, directly or indirectly, on membrane-remodeling processes involved in endothelial migration, sprouting, and lumen formation. However, the specific sequence of events following LF-EMF exposure has not yet been experimentally established.

**Figure 1:**
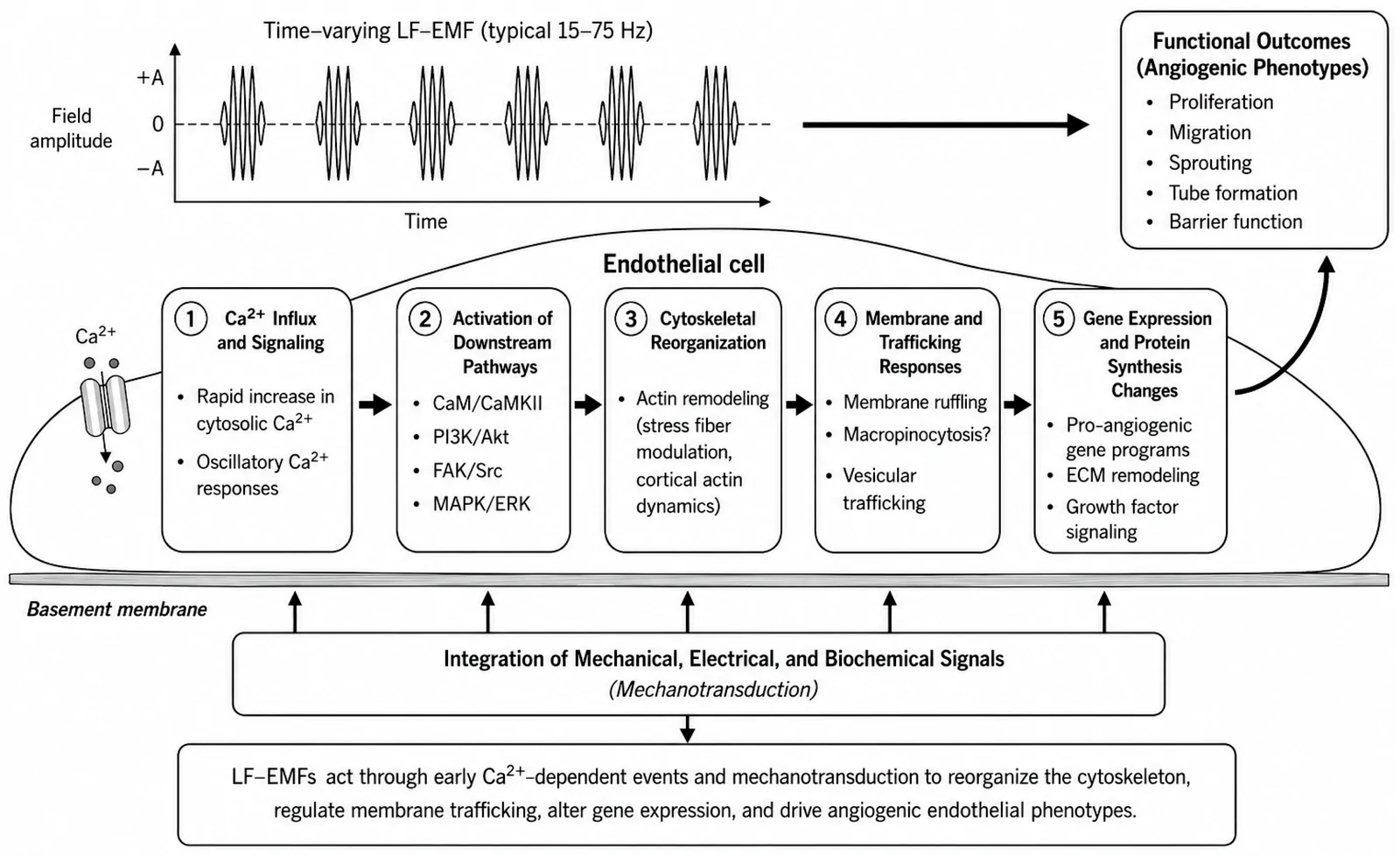
Proposed Mechanistic Framework for LF-EMF-Induced Endothelial Responses. Schematic overview of proposed endothelial responses to time-varying low-frequency electromagnetic fields (LF-EMFs), typically within the 15–75 Hz range. Early biophysical events include rapid Ca^2+^ influx and oscillatory calcium signaling, which activate downstream signaling pathways including CaM/CaMKII, PI3K/Akt, FAK/Src, and MAPK/ERK. These signaling cascades promote cytoskeletal remodeling, membrane ruffling, vesicular trafficking, and gene expression changes associated with angiogenic activity. Integration of electrical, mechanical, and biochemical signaling through mechanotransduction contributes to functional endothelial phenotypes including proliferation, migration, sprouting, tube formation, and barrier regulation. The schematic also highlights vesicular trafficking processes, including macropinocytosis, as potential contributors to LF-EMF-induced endothelial responses. The role of macropinocytosis proposed herein represents a hypothetical mechanistic model and has not yet been experimentally demonstrated.

LF-EMFs do not necessarily directly stimulate angiogenesis but instead perturb membrane-associated signaling pathways that endothelial cells already employ to integrate mechanical, electrical, and biochemical information. Collectively, these findings are consistent with a working model in which LF-EMFs may function as modulators of endothelial phenotype, influencing interconnected processes including calcium signaling, cytoskeletal dynamics, growth factor signaling, and vesicular trafficking pathways required for endothelial morphogenesis (Figure 1).

Throughout this review, experimentally demonstrated LF-EMF responses are distinguished from mechanistic hypotheses inferred through integration of historical ultrastructural observations with contemporary endothelial cell biology. Collectively, the available literature appears to support that LF-EMFs influence calcium signaling, nitric oxide production, and selected downstream signaling pathways in endothelial cells. In contrast, the proposed involvement of membrane trafficking, VEGFR2 internalization, and macropinocytosis represents an integrative mechanistic hypothesis based on combining historical ultrastructural observations with subsequent advances in endothelial cell biology rather than direct experimental demonstration following LF-EMF exposure, as detailed below.

### 3.4. Reinterpretation of Early Ultrastructural Observations: Macropinocytosis and Membrane Trafficking

Transmission electron microscopy studies documented vesicular structures, membrane ruffling, and vacuole formation during endothelial sprouting and lumen formation following LF-EMF exposure [10]. Although these features were carefully documented, they were not interpreted mechanistically at the time.

**Figure 2:**
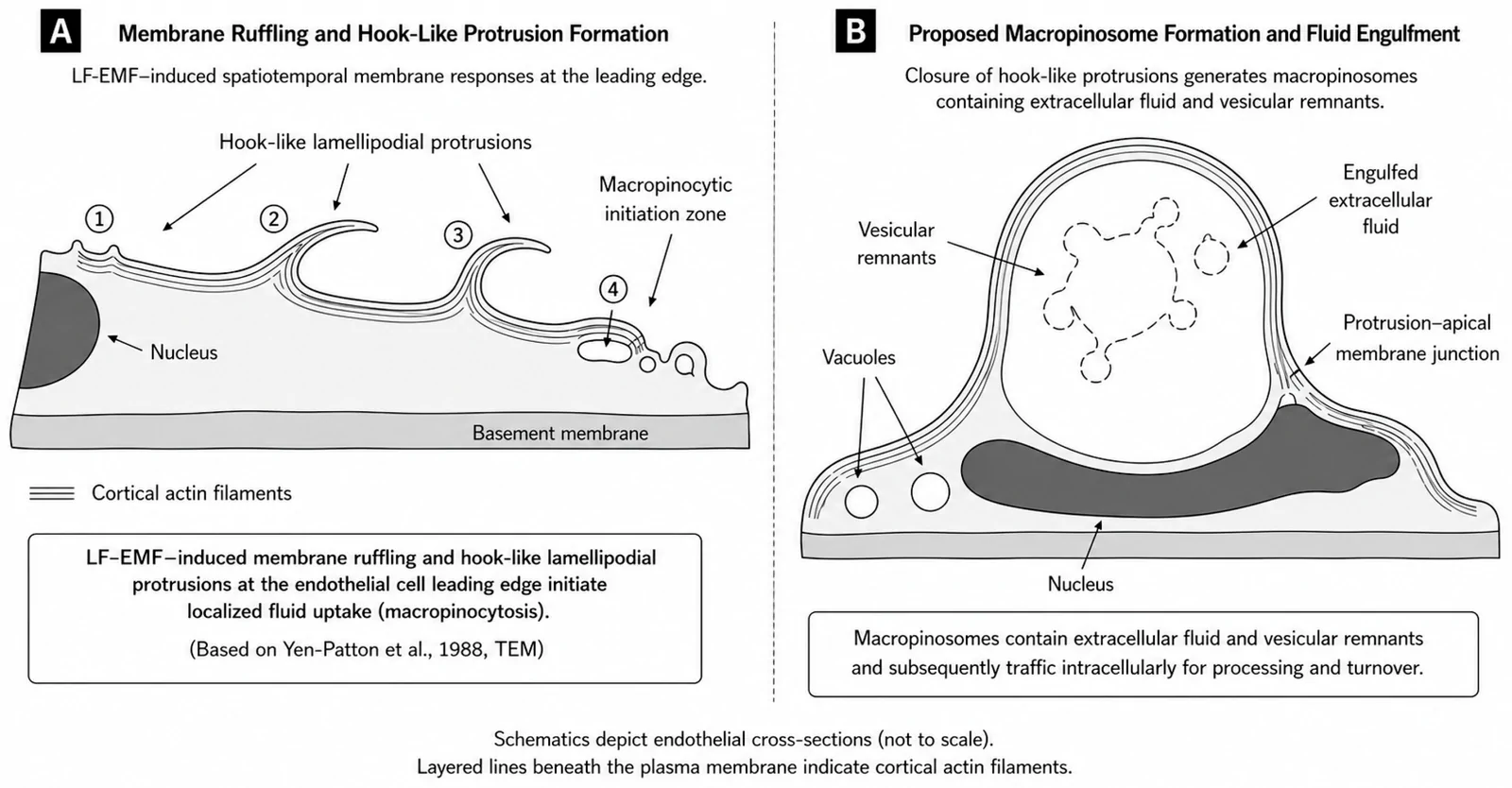
Early Membrane Responses to LF-EMFs: Membrane Ruffling and Macropinocytosis. Schematic interpretation of endothelial ultrastructural changes associated with low-frequency electromagnetic field (LF-EMF) exposure, based on transmission electron microscopy observations [10]. Panel (**A**): LF-EMF-associated membrane ruffling and hook-like lamellipodial protrusions initiate localized extracellular fluid engulfment, features that are consistent with but not diagnostic of early macropinocytosis. Early stages of protrusion formation and fluid engulfment are labeled numerically. Panel (**B**): closure and fusion of protrusions generate macropinosome-like structures containing extracellular fluid and vesicular remnants, with formation of a protrusion–apical membrane junction. These processes may contribute to endothelial membrane remodeling and early vascular lumen formation during angiogenesis. The proposed role of macropinocytosis represents a hypothetical mechanistic model based on reinterpretation of historical ultrastructural observations and has not yet been experimentally demonstrated following LF-EMF exposure.

Advances in endothelial cell biology now suggest that these ultrastructural features are consistent with vesicular trafficking processes associated with macropinocytosis, a pathway increasingly recognized as an important regulator of endothelial morphogenesis [28,29] (Figure 2).

An additional observation from the original PEMF studies provides further, albeit indirect, support for this interpretation. Endothelial cells exposed to PEMFs rapidly developed a distinctive sprouting morphology characterized by elongated cells, prominent membrane protrusions, and mycelial-like intercellular networks throughout otherwise confluent monolayers. After only 5 h of exposure, HUVEC exhibited approximately 20–30-fold more sprouting cells than parallel controls, while BAEC demonstrated approximately 10–15-fold increases. Importantly, this sprouting phenotype preceded overt vascular tube formation and represented the earliest visible cellular response to LF-EMF exposure.

Although the molecular basis of this sprouting phenotype was unknown in 1988, subsequent advances in endothelial biology have shown that angiogenic sprouting is initiated by dynamic membrane protrusions, including lamellipodia and membrane ruffles, which drive endothelial migration. These same membrane ruffles are now recognized as the sites from which macropinosomes arise during VEGFR2 signaling. Consequently, the dramatic increase in sprouting morphology is consistent with enhanced membrane dynamics that may precede macropinocytic receptor trafficking [10]. While this observation cannot establish that macropinocytosis occurred, it further strengthens the biological plausibility of the proposed mechanism.

Subsequent studies have demonstrated that VEGFR2 signaling is critically regulated by receptor internalization and intracellular trafficking. In particular, VEGF-A-induced VEGFR2 uptake occurs predominantly through CDC42-dependent macropinocytosis, a process required for sustained angiogenic signaling and endothelial morphogenesis [29]. Beyond receptor internalization, intracellular membrane trafficking is now recognized as an essential regulator of endothelial migration, sprouting, vacuole formation, and lumen formation during angiogenesis [30,31,32]. Viewed in this context, the membrane ruffling, intracellular vesicles, vacuoles, and lumen-forming structures reported by Yen-Patton et al. can be reinterpreted as manifestations of enhanced membrane remodeling accompanying angiogenic morphogenesis [10].

Nevertheless, no molecular markers of macropinocytosis, receptor trafficking, or endosomal signaling were examined in the original LF-EMF studies. Consequently, the proposed involvement of macropinocytosis should be regarded as a biologically plausible mechanistic hypothesis rather than an experimentally established pathway. Taken together, however, the historical ultrastructural observations, the early sprouting phenotype, and subsequent advances in endothelial membrane trafficking provide a coherent conceptual framework that integrates nearly four decades of endothelial angiogenesis research. Future studies combining live-cell imaging with molecular markers of membrane ruffling, macropinosome formation, and VEGFR2 trafficking will be required to determine whether LF-EMF exposure directly engages this pathway during endothelial angiogenesis.

## 4. Discussion

### 4.1. Parameter Optimization Challenges

LF-EMFs comprise a broad class of time-varying electromagnetic exposures, including both pulsed electromagnetic fields (PEMFs) and continuous sinusoidal fields, characterized by diverse waveform shapes, frequencies, and amplitudes. These fields can be applied noninvasively via electromagnetic induction, generating secondary electric fields in tissues without requiring implanted electrodes and thereby minimizing incidental risks such as microbial contamination.

According to the International Telecommunication Union (ITU), electromagnetic fields below 300 Hz fall within the extremely low frequency (ELF) and super low frequency (SLF) bands, encompassing the LF-EMF exposures commonly used in biological systems [24,33,34]. These frequencies have attracted considerable scientific interest because of both their therapeutic potential and ongoing public-health debate. Notably, extremely low-frequency magnetic fields were classified by the International Agency for Research on Cancer (IARC) as Group 2B (“possibly carcinogenic to humans”), based primarily on limited epidemiological evidence relating to childhood leukemia rather than experimental evidence of endothelial dysfunction or angiogenesis [35]. Consequently, understanding the biological mechanisms through which LF-EMFs interact with living systems remains important from both therapeutic and health-risk perspectives. Most prior studies have focused on LF-EMF exposures developed for orthopedic and musculoskeletal applications, although considerable variability in waveform design, frequency, magnetic field strength, and treatment protocols has limited direct comparison among studies [36,37].

LF-EMF waveforms can be tuned through pulse period, burst structure, amplitude, and number of pulses per burst [37]. Historically, commonly evaluated signals include 15 Hz pulse trains and 72 Hz repetitive single-pulse modalities derived from early commercial devices (e.g., Electro-Biology, Inc., Parsippany, NJ, USA), as well as lower-frequency (e.g., 2 Hz) repetitive pulse systems [37]. These pulsed waveforms typically induce electric fields of ~1–100 mV/cm and exhibit quasi-rectangular or trapezoidal profiles, often with reverse-polarity excursions. In contrast, sinusoidal LF-EMFs generate continuous oscillatory electric fields with a narrow spectral distribution, complementing the broadband spectral content of pulsed signals.

Despite this flexibility, relatively few studies have systematically explored the LF-EMF parameter space, with many investigations relying on fixed exposure conditions derived from commercial orthopedic devices or conventional 50/60 Hz power-frequency exposure systems. The available waveform space for LF-EMF alone has been estimated to approach ~10^15^ distinct configurations, a complexity further expanded by inclusion of sinusoidal and other continuous waveforms [38]. Application of Design of Experiments (DoE) methodologies could enable systematic identification of biologically active parameter regimes and transition LF-EMF research toward predictive frameworks [39]. Improved standardization and reporting of exposure parameters, including clear distinction between pulsed and sinusoidal waveforms, is essential for reproducibility, cross-study comparison, and regulatory evaluation of electromagnetic therapeutic devices.

### 4.2. Strengths, Limitations, and Outstanding Questions

A major strength of the current literature is the broad convergence of endothelial responses observed across multiple independent model systems and exposure paradigms. Studies employing both pulsed electromagnetic fields (PEMFs) and sinusoidal low-frequency electromagnetic fields (LF-EMFs) have reported enhancement of endothelial cell proliferation, migration, sprouting, tube formation, and vascular remodeling under a broad range of experimental conditions. Although these studies differ substantially in waveform, frequency, magnetic field strength, exposure duration, and experimental model, many report qualitatively similar pro-angiogenic endothelial responses. This recurring pattern suggests that endothelial cells are responsive to LF-EMF stimulation under multiple experimental conditions, while also indicating that the magnitude and specific characteristics of the response depend strongly on exposure parameters and biological context. Accordingly, the available evidence supports the existence of a general endothelial response to LF-EMFs rather than a uniform effect across all exposure paradigms. Although the majority of studies reviewed report pro-angiogenic endothelial responses, substantial variability in the magnitude of these responses has been observed, suggesting that biological outcomes depend strongly on exposure parameters, cell type, and experimental context.

Despite this response consistency, several important limitations constrain mechanistic interpretation. The available literature remains relatively small, and many studies employ substantially different exposure parameters, including waveform characteristics, frequency, magnetic field strength, exposure duration, and dosimetry methodologies. The continued development and adoption of standardized exposure platforms that provide rigorous dosimetric characterization, well-defined magnetic-field homogeneity, precise environmental control, reproducible sham exposure conditions, and comprehensive reporting of exposure parameters are required. Recent methodological advances demonstrate that carefully engineered exposure systems incorporating validated dosimetry, temperature regulation, and standardized quality-control procedures can substantially improve experimental reproducibility and facilitate meaningful comparison among independent studies [40,41]. Adoption of similarly standardized platforms for endothelial angiogenesis research should strengthen mechanistic interpretation, improve interlaboratory reproducibility, and facilitate translation of LF-EMF angiogenesis research from exploratory observations toward reproducible, hypothesis-driven investigations. Variability in endothelial cell sources, culture conditions, and angiogenesis assays further complicates direct comparison among studies. Moreover, relatively few investigations have systematically explored exposure-response relationships or independently replicated specific parameter combinations across laboratories. As a result, it remains difficult to determine whether observed biological effects arise from common underlying mechanisms or from distinct responses elicited by different exposure conditions.

Interpretation of the ultrastructural observations discussed in this review should likewise be considered within the context of these limitations. Although the membrane ruffling, protrusive structures, vacuoles, and lumen-associated membrane dynamics reported by Yen-Patton et al. (1988) are consistent with contemporary descriptions of macropinocytosis and vesicular trafficking, these observations were not originally examined using molecular or fluorescence-based markers capable of definitively identifying specific trafficking pathways, such as VEGFR2 internalization, CDC42-dependent macropinocytosis, Rab5-positive early endosomes, fluorescent dextran uptake, or actin-dependent membrane remodeling [10,29,32,42]. Consequently, the mechanistic framework proposed here should be viewed as a testable hypothesis rather than a definitive explanation. Alternative membrane-remodeling processes associated with endothelial morphogenesis may contribute to the observed ultrastructural features, and direct experimental validation will be required to distinguish among these possibilities.

Several outstanding questions therefore emerge. Do LF-EMFs directly influence membrane trafficking pathways, or are trafficking changes secondary to alterations in calcium signaling and growth factor responsiveness? Does electromagnetic field stimulation alter VEGFR2 internalization, endosomal signaling, or receptor recycling dynamics? Are endothelial responses governed primarily by magnetic field parameters, induced electric fields, or interactions between the two? Finally, to what extent are angiogenic responses waveform-specific, and can systematic optimization identify exposure parameters that reproducibly maximize biological activity? Addressing these questions will be essential for transforming the current descriptive literature into a predictive mechanistic framework capable of supporting translational bioelectromagnetic applications.

### 4.3. Future Research Directions

Future studies should focus on experimentally testing the mechanistic hypotheses identified above using model systems capable of bridging reductionist endothelial cell cultures and physiologically integrated vascular tissues. In particular, approaches combining quantitative angiogenesis assays with direct measurements of membrane trafficking, receptor internalization, and endothelial signaling may help determine whether the proposed macropinocytosis framework contributes to LF-EMF-induced vascular remodeling. Such studies will also be essential for distinguishing the relative contributions of magnetic field parameters, induced electric fields, and waveform characteristics to endothelial angiogenic responses.

Future studies should also seek to establish quantitative relationships between electromagnetic field exposure parameters and the cellular processes governing endothelial cell remodeling. Integrating electromagnetic dosimetry with measurements of ion transport, membrane dynamics, receptor trafficking, and cytoskeletal remodeling may help bridge the gap between physical field parameters and the molecular mechanisms underlying angiogenesis.

Although this review intentionally focuses on endothelial biology because angiogenesis is ultimately an endothelial-driven process, successful vascular remodeling also involves fibroblasts, pericytes, inflammatory cells, and extracellular matrix remodeling. LF-EMFs have been reported to influence several of these cell types, but those effects are beyond the scope of the present review. Additionally, the effects of LF-EMFs on cultured lymphatic endothelial cells have not been systematically investigated. Comparative studies examining vascular and lymphatic endothelial cells under identical exposure conditions would help determine whether LF-EMF responses are conserved across endothelial lineages or reflect differences in VEGFR2- and VEGFR3-dependent signaling pathways. Such studies could provide important insight into the specificity of proposed membrane trafficking and receptor signaling mechanisms underlying endothelial responses to LF-EMFs.

Future mechanistic studies should also examine endothelial responses within multicellular tissue environments, including wound-healing models containing endothelial cells, fibroblasts, immune cells, and extracellular matrix. Such systems could determine whether LF-EMF-induced endothelial responses are preserved, modified, or indirectly mediated by neighboring cell populations. Because fibroblast proliferation and migration do not necessarily predict endothelial angiogenesis, results from nonvascular cell types should be treated as complementary rather than directly transferable evidence. Addressing these mechanistic questions will likely require experimental platforms that bridge reductionist endothelial cell cultures and physiologically integrated vascular tissues. Intermediate in vivo angiogenesis models offer a particularly attractive approach because they preserve experimental accessibility while incorporating essential features of tissue organization and vascular function. Despite substantial progress in endothelial cell culture systems, translation of LF-EMF angiogenesis research to intact organisms presents important experimental and biophysical challenges. Exposure conditions that can be tightly controlled in endothelial monolayers become more difficult to reproduce in vivo because of tissue heterogeneity, animal movement, complex electrical properties, and limitations in electromagnetic field uniformity [24,33]. In addition, transition from planar endothelial cell cultures to three-dimensional tissues complicates interpretation of magnetic-field versus induced electric-field contributions, as tissue geometry, conductivity, and vascular orientation influence local electrical gradients [27].

One particularly promising intermediate platform is the chicken chorioallantoic membrane (CAM) assay, a highly vascularized extraembryonic membrane widely used for studies of angiogenesis, vascular remodeling, and biomaterial integration [20]. The CAM provides a relatively thin, planar-like vascular network that preserves intact blood flow, extracellular matrix interactions, and multicellular tissue organization while retaining experimental accessibility comparable to endothelial monolayer systems. This configuration permits direct imaging and quantitative assessment of vascular branching, sprouting, vessel density, and network remodeling under controlled LF-EMF exposure conditions. Importantly, the geometric simplicity of the CAM should facilitate mechanistic investigation of magnetic-field effects and secondary induced electric fields generated through electromagnetic induction.

Complementary progression toward mammalian systems may be achieved using retinal angiogenesis models, particularly neonatal mouse retinal vascular development assays. In a neonatal retinal angiogenesis model, LF-EMF exposure could be delivered using a calibrated coil system sized to encompass the animal and generate a reproducible magnetic field across the body. A Helmholtz configuration would be appropriate for studies requiring relatively uniform exposure, whereas gradient-producing geometries could be used to examine spatially dependent responses. Experimental design would require sham-exposed controls and careful monitoring of field uniformity, induced electric fields, temperature, animal movement, and exposure duration. Following exposure, retinal whole mounts could be analyzed for radial vascular expansion, vessel density, branching, tip-cell activity, and avascular area. This system permits quantitative analysis of endothelial sprouting, tip-cell behavior, vascular branching, and lumen formation within an intact mammalian neurovascular environment [43]. Similar to the CAM assay, the planar organization of the retinal vasculature provides advantages for evaluating field orientation effects, induced electrical gradients, and endothelial signaling responses under controlled exposure conditions.

Together, CAM and retinal vascular models provide attractive intermediate platforms bridging reductionist endothelial cell monolayer systems and more complex mammalian disease models. Their use may enable direct testing of emerging mechanistic hypotheses, including the proposed involvement of membrane trafficking and macropinocytosis in LF-EMF-induced endothelial remodeling. Ultimately, integration of standardized exposure paradigms with physiologically relevant angiogenesis models will be essential for establishing predictive mechanistic frameworks and advancing translational applications of bioelectromagnetic field stimulation.

## 5. Conclusions

Collectively, the available literature indicates that LF-EMFs can influence multiple stages of endothelial angiogenesis under diverse experimental conditions, encompassing both pulsed and sinusoidal exposure paradigms. Reported endothelial responses include enhanced proliferation, migration, sprouting, tube formation, and vascular remodeling. Across diverse experimental systems, many reported endothelial cell responses are associated with signaling pathways involving calcium regulation, growth factor responsiveness, nitric oxide production, and cytoskeletal reorganization, supporting the possibility that time-varying electromagnetic stimulation can influence fundamental processes underlying vascular morphogenesis.

A central theme emerging from this review is that membrane trafficking processes may represent an underappreciated mechanistic component of LF-EMF-induced endothelial remodeling. Reinterpretation of historical ultrastructural observations within the framework of contemporary endothelial cell biology suggests that macropinocytosis, receptor trafficking, and vesicular dynamics may contribute to the endothelial responses observed following LF-EMF exposure. Although this interpretation remains hypothetical and requires direct experimental validation, it provides a biologically plausible framework linking electromagnetic field stimulation to endothelial morphogenesis and angiogenic behavior. Establishing a predictive mechanistic framework linking defined electromagnetic exposure parameters to endothelial signaling will be essential for translating LF-EMF therapies from empirical observations toward rationally designed regenerative medicine and bioelectromagnetic applications.

## Figures and Tables

**Table 1:** Representative Studies of LF-EMF-Induced Endothelial Cell Angiogenesis.

Study	Model System	EMF Parameters	Exposure	Principal Biological Findings	Research Advancement
**Foundational endothelial angiogenesis studies (Discovery phase)**					
Yen-Patton et al., 1988 [10]	HUVEC, BAEC	1.2 mT, 15 Hz	Hours–days	Migration, proliferation, sprouting, tube formation	First demonstration of LF-PEMF-induced endothelial angiogenesis
Chung-Welch et al., 1989 [13]	BPMEC	1.2 mT, 15 Hz	Hours–days	Tube formation	Established endothelial response specificity
Goodman et al., 1993 [14]	HUVEC	2 mT, 25 Hz sinusoidal	Hours	Cell migration	Independent confirmation of endothelial migratory responses
**Mechanistic expansion phase (2001–2019)**					
Tepper et al., 2004 [11]	HUVEC	~1.2 mT, 15 Hz	Days	Sprouting and tube formation	Implicated FGF-2 signaling in LF-PEMF-induced angiogenesis
Delle Monache et al., 2008 [12]	HUVEC	1 mT, 50 Hz sinusoidal	Hours	Tube formation; nitric oxide signaling; Calcium signaling, cytoskeletal remodeling.	Linked angiogenesis to VEGFR2/nitric oxide signaling
Hopper et al., 2009 [15]	HUVEC	1.8 mT, 15 Hz	8 h	Proliferation; signaling types	Identified autocrine and paracrine signaling
Cheng et al., 2017 [16]	HUVEC	2.25 mT, 50 Hz	24 h	Akt/mTOR signaling	Identified Akt/mTOR signaling
Prucha et al., 2019 [17]	HUVEC	4 mT, 72 Hz	24 h	Enhanced proliferation	Demonstrated waveform-dependent endothelial responses.
Xu et al., 2019 [18]	HUVEC	200 mT, 4 Hz sinusoidal	Hours	AMPK activation	Identified AMPK-mediated metabolic signaling
**Modern molecular mechanism phase (2020–2025)**					
Yang et al., 2024 [19]	HUVEC	1 mT, 40 Hz	24–72 h	Metabolic reprogramming; mitochondrial fission	Linked angiogenesis to mitochondrial remodeling
Font et al., 2025 [20]	HMEC-1, chicken CAM	13.5 mT, 10 or 60 Hz sinusoidal	20 min	Nitric oxide signaling; angiogenesis	Integrated NO signaling with in vitro and in vivo findings

Representative studies investigating the effects of low-frequency electromagnetic fields (LF-EMFs) on endothelial angiogenesis. The Research advancement column summarizes each study’s principal contribution to the evolution of the field, from initial phenotypic observations to mechanistic understanding. Sinusoidal LF-EMF exposures are explicitly indicated in the table; all other listed studies employed pulsed electromagnetic field (PEMF) waveforms.

## Data Availability

No new data were created or analyzed in this study. Data sharing is not applicable to this article.

## Abbreviations

The following abbreviations are used in this manuscript:

Akt (PKB)	Protein kinase B
AMPK	AMP-activated protein kinase
BAEC	Bovine aortic endothelial cells
BPMEC	Bovine pulmonary microvascular endothelial cells
BrdU	Bromodeoxyuridine
Ca^2+^	Calcium ion
CaM	Calmodulin
CaMKII	Calcium/calmodulin-dependent protein kinase II
CAM	Chorioallantoic membrane
CDC42	Cell division control protein 42 homolog
CD31 (PECAM-1)	Platelet endothelial cell adhesion molecule-1
DoE	Design of Experiments
ECM	Extracellular matrix
ELF	Extremely low frequency
EMF	Electromagnetic field
FAK	Focal adhesion kinase
FGF-2	Fibroblast growth factor-2
HMEC-1	Human microvascular endothelial cell line-1
HUVEC	Human umbilical vein endothelial cells
IARC	International Agency for Research on Cancer
ICR	Ion cyclotron resonance
IL	Interleukin
ITU	International Telecommunication Union
ITU-R	International Telecommunication Union Radiocommunication Sector
LF-EMF	Low-frequency electromagnetic field
MAPK	Mitogen-activated protein kinase
PEMF	Pulsed electromagnetic field
